# Lateralized Resting-State Functional Brain Network Organization Changes in Heart Failure

**DOI:** 10.1371/journal.pone.0155894

**Published:** 2016-05-20

**Authors:** Bumhee Park, Bhaswati Roy, Mary A. Woo, Jose A. Palomares, Gregg C. Fonarow, Ronald M. Harper, Rajesh Kumar

**Affiliations:** 1 Department of Anesthesiology, University of California Los Angeles, Los Angeles, California, United States of America; 2 UCLA School of Nursing, University of California Los Angeles, Los Angeles, California, United States of America; 3 Division of Cardiology, University of California Los Angeles, Los Angeles, California, United States of America; 4 Brain Research Institute, University of California Los Angeles, Los Angeles, California, United States of America; 5 Department of Neurobiology, University of California Los Angeles, Los Angeles, California, United States of America; 6 Department of Radiological Sciences, University of California Los Angeles, Los Angeles, California, United States of America; 7 Department of Bioengineering, University of California Los Angeles, Los Angeles, California, United States of America; Nathan Kline Institute and New York University School of Medicine, UNITED STATES

## Abstract

Heart failure (HF) patients show brain injury in autonomic, affective, and cognitive sites, which can change resting-state functional connectivity (FC), potentially altering overall functional brain network organization. However, the status of such connectivity or functional organization is unknown in HF. Determination of that status was the aim here, and we examined region-to-region FC and brain network topological properties across the whole-brain in 27 HF patients compared to 53 controls with resting-state functional MRI procedures. Decreased FC in HF appeared between the caudate and cerebellar regions, olfactory and cerebellar sites, vermis and medial frontal regions, and precentral gyri and cerebellar areas. However, increased FC emerged between the middle frontal gyrus and sensorimotor areas, superior parietal gyrus and orbito/medial frontal regions, inferior temporal gyrus and lingual gyrus/cerebellar lobe/pallidum, fusiform gyrus and superior orbitofrontal gyrus and cerebellar sites, and within vermis and cerebellar areas; these connections were largely in the right hemisphere (p<0.005; 10,000 permutations). The topology of functional integration and specialized characteristics in HF are significantly changed in regions showing altered FC, an outcome which would interfere with brain network organization (p<0.05; 10,000 permutations). Brain dysfunction in HF extends to resting conditions, and autonomic, cognitive, and affective deficits may stem from altered FC and brain network organization that may contribute to higher morbidity and mortality in the condition. Our findings likely result from the prominent axonal and nuclear structural changes reported earlier in HF; protecting neural tissue may improve FC integrity, and thus, increase quality of life and reduce morbidity and mortality.

## Introduction

Heart failure (HF) patients show multiple autonomic, sensorimotor, mood, and cognitive deficits [1–4], which may originate from hypoxia/ischemia-induced brain injury by low cardiac output and sleep-disordered breathing, subsequent to cerebral hypo-perfusion in the condition [5–7]. Short-term memory loss is one of the most common cognitive changes reported in HF, with an incidence of ranging from 23–80% of HF cases (a risk of nearly twice that of healthy/non-HF patients) [8]. Also, executive decision making function is another serious cognitive deficit, affecting ~24% HF patients [9]. Persons with short-term memory loss and executive function deficit have impaired ability to learn and carry out important self-management strategies, such as to accurately and appropriately follow dietary and medication regimens, recognize symptoms associated with deteriorating health, and when to communicate with their health care provider [10, 11]. With the loss of memory and ability to learn how to self-manage their HF and decide upon needed communication with health care provider, there is increased risk for HF exacerbations and associated increased morbidity and mortality in this serious medical condition [1, 10, 11]. Similarly, high incidence of mood issues, including depression (40–60%) and anxiety (up to 45%) [2, 12] in HF patients may interfere with day-to-day self-management activity, and contribute to increased morbidity and mortality.

Brain structural injury appears in multiple brain regions serving autonomic, sensorimotor, mood, and cognitive functions based on various magnetic resonance imaging (MRI) procedures, including high-resolution T1-weighted imaging, T2-relaxometry, and diffusion tensor imaging (DTI) [13–16]. The structural impairments lead to aberrant functional MRI responses to autonomic challenges, including the Valsalva maneuver and cold pressor stimuli [3, 4, 17], and may also alter overall spontaneous functional organization, labeled “resting-state functional connectivity” (FC). It is reasonable to assume that impaired resting-state functional organization contributes to momentary neuropsychologic and physiologic pathology in HF, and may exacerbate the potential for further injury. However, whole-brain structural interactions during resting states (termed connection “weights” among brain regions) and coordination of these interactions (*i*.*e*., brain network organization) remain unclear in HF.

Resting-state functional MRI (rs-fMRI) procedures have been used to investigate region-to-region FC, a term which refers to temporal statistical dependency between neuronal activities of anatomically-distinct brain regions [18]. The procedure identifies synchronized spontaneous low-frequency (<0.1 Hz) fluctuation of blood-oxygen-level-dependent (BOLD) signals across the brain in the resting-state [19–21], which appear as consistent patterns across healthy subjects [22–25]. Resting-state FC procedures have been applied widely in various functional brain network studies, ranging from psychiatric to neurological conditions [26], and as well as in evaluation of human brain functions [27–29]. Since rs-fMRI FC procedures are used to discriminate healthy controls from patients (e.g., with stroke) [30], FC can be a potential biomarker and may be useful in assessing interactions of functional brain networks and coordination of these interactions in HF population.

Network-level approaches, based on graph theory, can describe the organizational properties of functional brain networks [31, 32]. A brain network can be modeled graphically, often called a “brain graph”, which consists of a set of nodes (brain regions) and edges (connectivity between nodes) [31, 33, 34]. Network-level approaches suggest that human brain networks are organized into modular systems, which are characterized by efficient integration of segregated brain regions through short paths, with low wiring costs, consisting of a few densely-connected core regions in the whole-brain [31, 32, 35]. These organizational brain attributes have been found in both anatomical, using DTI or cortical thickness assessments [36–40], and functional networks, using MEG, EEG, or fMRI [41–45]. It has been suggested that cognitive processing is based on high global efficiency in brain networks to efficiently integrate neural information across whole-brain sites [34], where some hubs (e.g., brain regions concentrated by a large number of connections with the rest of whole-brain) play a pivotal role showing high cost for conveying neural information and are especially vulnerable to aberrant disease conditions [46]. Various disease conditions, including Schizophrenia, Alzheimer’s disease, and Parkinson’s disease [46], show that pathological brain areas are significantly concentrated in such hub sites playing critical role in normal functional brain network, although cortical hubs are differently lesioned in each disorder. Thus, network-level approaches may also yield important macroscopic or topological alterations on functional brain network in HF patients as well.

Our aim was to investigate functional interactions and organizational properties across the whole-brain in HF patients over age- and gender-comparable controls using FC and basic network-level approaches. We hypothesized that HF patients would show intrinsically-abnormal brain FC and functional coordination among the regions serving multiple autonomic, sensori-motor, mood, and cognitive roles in HF.

## Materials and Methods

### Subjects

We investigated 27 hemodynamically-optimized (drug dosages were titrated to reach targeted hemodynamic goals) HF patients and 53 age- and gender-comparable healthy controls. Demographic and clinical data of all HF patients and controls are summarized in Table 1. All HF patients were recruited from the Ahmanson-University of California at Los Angeles (UCLA) Cardiomyopathy Center. The diagnosis of HF was based on national diagnostic criteria [47], and all subjects included in this study were with NYHA Functional Class II at the time of MRI [48]. All HF patients enrolled here were between 30–66 years of age. Lower age was chosen to minimize developmental process and higher age was chosen to reduce aging effect. All HF patients were without any history of drug abuse, valvular congenital heart defects, pregnancy induced cardiomyopathy, no previous history of stroke or carotid vascular disease, and head injury. All HF patients were treated with guideline-directed medical therapy, including angiotensin receptor blockers or angiotensin-converting enzyme inhibitors, beta blockers, and diuretics, and were stabilized for hemodynamics and body-weight for at least six months prior to participation in MRI studies.

**Table 1:** Demographic, clinical, and sleep variables of HF and control subjects.

Variables	HF (n = 27)	Controls (n = 53)	P-value
Age range (y)	40–66	42–66	−
Age (mean ± SD, yrs)	55.3 ± 7.9	52.7 ± 6.2	0.11
Sex (Male:Female)	20:7	36:17	0.57
BMI (mean ± SD, kg/m^2^)	27.9 ± 5.5	25.4 ± 3.5	0.01
Handedness	2 Left; 24 Right; 1 Ambidextrous	10 Left; 42 Right; 1 Ambidextrous	−
Ethnicity	1 Asian; 17 White; 2 Hispanic; 6 African-American; 1 Armenian	14 Asian; 25 White; 8 Hispanic; 3 African-American; 1 White-Asian; 1 Hispanic-White; 1 El Salvador-Hispanic	−
PSQI (mean ± SD)	7.2 ± 3.9	4.0 ± 2.6	<0.001
ESS (mean ± SD)	5.4 ± 3.2 (n = 26)	8.0 ± 4.2	0.0027
BDI-II (mean ± SD)	10.3 ± 7.1	3.9 ± 4.1	<0.001
BAI (mean ± SD)	9.5 ± 8.0	3.7 ± 4.7	<0.001
LVEF (mean ± SD)	28.0 ± 9.2	−	−
Global MoCA scores (mean ± SD)	24.9 ± 3.4 (n = 10)	27.7 ± 1.9 (n = 16)	0.01
MoCA: Visuospatial (mean ± SD)	3.3 ± 1.4 (n = 10)	4.4 ± 0.6 (n = 16)	0.04
MoCA: Naming (mean ± SD)	3.0 ± 0.0 (n = 10)	2.8 ± 0.6 (n = 16)	0.10
MoCA: Attention (mean ± SD)	5.5 ± 0.8 (n = 10)	5.6 ± 0.6 (n = 16)	0.83
MoCA: Language (mean ± SD)	2.2 ± 0.8 (n = 10)	2.7 ± 0.8 (n = 16)	0.14
MoCA: Abstraction (mean ± SD)	2.0 ± 0.0 (n = 10)	2.0 ± 0.0 (n = 16)	1.0
MoCA: Delayed recall (mean ± SD)	2.9 ± 1.8 (n = 10)	4.3 ± 0.9 (n = 16)	0.04
MoCA: Orientation (mean ± SD)	6.0 ± 0.0 (n = 10)	6.0 ± 0.0 (n = 16)	1.0

BMI, Body mass index; ESS, Epworth sleepiness scale; PSQI, Pittsburgh sleep quality index; BDI-II, Beck depression inventory II; BAI, Beck anxiety inventory; LVEF, Left ventricular ejection fraction; MoCA, Montreal Cognitive Assessment; SD, Standard deviation.

Control subjects were recruited through advertisements at the UCLA campus and Los Angeles area. All control subjects were in good health, without any clinical history of cardiovascular, stroke, respiratory, neurological, or psychiatric disorders that may introduce brain changes.

Both HF patients and controls were excluded from the study if they were claustrophobic, carrying non-removable metal, such as embolic coils, pacemakers/implantable cardioverter-defibrillators, stents, or with body weight more than 125 kg (the last, a scanner limitation). All subjects gave written and informed consent before data acquisition and study protocol was approved by the Institutional Review Board at the UCLA.

### Mood and sleep examination

Beck Depression Inventory II (BDI-II) was used to assess depressive symptoms, and Beck Anxiety Inventory (BAI) was used to examine anxiety symptoms in HF patients and controls [49, 50]. Both BDI-II and BAI are self-administered questionnaires (21 questions; each score ranged from 0–3) with total scores ranging from 0–63, based on mood or anxiety symptoms.

Sleep quality and daytime sleepiness were evaluated in HF patients and controls. We used the Pittsburgh Sleep Quality Index (PSQI) [51]and Epworth Sleepiness Scale (ESS) [52] to examine sleep quality and day time sleepiness, respectively.

### Cognition assessment

The Montreal Cognitive Assessment (MoCA) test was used for cognitive assessment. The test contains various cognitive domains, including attention, executive functions, memory, language, visuo-constructional skills, conceptual thinking, calculations, and orientation [53].

### Magnetic resonance imaging

Brain imaging of HF patients and controls was performed using a 3.0-Tesla MRI scanner (Siemens, Magnetom Tim-Trio, Erlangen, Germany). Foam pads were used on either side of the head to reduce head motion-related artifacts during scanning. Rs-fMRI data were acquired with an echo planar imaging based BOLD sequence in the axial plan [repetition time (TR) = 2000 ms; echo time (TE) = 30 ms; flip angle (FA) = 90°; field-of-view (FOV) = 230×230 mm^2^; matrix size = 64×64; voxel size = 3.59×3.59×4.5 mm^3^; volumes = 59], while participants lay resting with eyes open, without focusing on specific thoughts, and no sleeping during about 2 minutes. High resolution T1-weighted images were collected from each subject using a magnetization prepared rapid acquisition gradient-echo pulse sequence (TR = 2200 ms; TE = 2.2, 2.34 ms; FA = 9°; FOV = 230×230 mm^2^; matrix size = 256×256, 320×320; voxel size = 0.9×0.9×1.0 mm^3^, 0.72×0.72×0.9 mm^3^). Proton-density (PD) and T2-weighted images were acquired in the axial plane, using a dual-echo turbo spin-echo pulse sequence (TR = 10,000 ms; TE1, 2 = 17, 134 ms; FA = 130°; matrix size = 256×256; FOV = 230×230 mm^2^; voxel size = 0.9×0.9×4.0 mm^3^).

### Data preprocessing

We used the statistical parametric mapping (SPM8, Wellcome Department of Cognitive Neurology, London, UK) [54] and MRIcroN software [55] for evaluation of images and rs-fMRI data preprocessing. High-resolution T1-weighted, PD-, and T2-weighted images of HF patients and controls were examined for any gross brain pathology, such as tumors, cysts, or major infarcts. Rs-fMRI data were also assessed for imaging or head motion-related artifacts before data processing. No subjects included in this study showed any serious visible brain pathology, head motion-related, or other imaging artifacts.

Resting-state fMRI data preprocessing steps included realignment of EPI brain volumes for removal of any potential head-motion, co-registration to T1-weighted images, and spatial normalization to a standard common space template using nonlinear transformation procedures. For rs-fMRI data analysis, we discarded the initial 3 brain volumes to avoid signal saturation issues, and used the remaining 56 volumes for remaining analysis. No spatial smoothing was performed on the rs-fMRI data to avoid inflation of local connectivity and clustering [56].

### Construction and analysis of functional network

Individual whole-brain FC was determined from regional mean fMRI time series, extracted from 116 distinct regions, as defined by automated anatomical labeling [57], that consists of 90 cerebral brain regions (45 sites in each hemisphere) and 26 cerebellar areas (9 regions in each hemisphere and 8 vermis sites), as described in Table 2. We applied the canonical signal processing procedures for calculating the rs-FC for each regional mean fMRI time series [58]. Each time series was band-pass filtered (0.009–0.08 Hz), and effects of six rigid motions, their first derivatives, and global signal changes in white matter, cerebrospinal fluid, and whole-brain were removed by regression. The first derivatives of the motion parameters were added in the statistical model to minimize signal changes from head-motion [59], which is often an issue in any rs-FC study [59–61]. We defined FC (edge) as an inter-regional correlation map among 116 preprocessed regional time series. We converted individual correlation maps into z-scored maps with Fisher’s r-to-z transformation to improve normality. We compared the z-scored maps edge-by-edge between HF patients and controls using analysis of covariance (ANCOVA), with age and gender included as covariates. We also examined relationships between each variable (BMI, LVEF, PSQI, ESS, BAI, or BDI-II) and functional connectivity. All rs-FC analyses were performed using MATLAB-based custom software.

**Table 2:** Sites with abbreviations corresponding to 116 brain regions.

Regions	Abbreviation	Regions	Abbreviation
Precental gyrus	PrCG	Supramarginal gyrus	SMG
Superior frontal gyrus (dorsolateral part)	SFGdor	Angular gyrus	ANG
Orbitofrontal gyrus (superior part)	OFGsup	Precuneus	PRCU
Middle frontal gyrus	MFG	Paracentral lobule	PCL
Orbitofrontal gyrus (middle part)	OFGmid	Caudate	CAU
Inferior frontal gyrus (opercular part)	IFGop	Putamen	PUT
Inferior frontal gyrus (triangular part)	IFGtr	Pallidum	PAL
Orbitofrontal gyrus (inferior part)	OFGinf	Thalamus	THL
Rolandic operculum	ROL	Heschl gyrus	HES
Supplementary motor area	SMA	Superior temporal gyrus	STG
Olfactory cortex	OLF	Temporal pole (superior part)	TPsup
Superior frontal gyrus (medial part)	SFGmed	Middle temporal gyrus	MTG
Orbitofrontal gyrus (medial part)	OFGmed	Temporal pole (middle part)	TPmid
Rectus	REC	Inferior temporal gyrus	ITG
Insula	INS	Cerebellar crus I	CRcr-I
Anterior cingulate cortex	ACC	Cerebellar crus II	CRcr-II
Middle cingulate cortex	MCC	Cerebellum III	CR-III
Posterior cingulate cortex	PCC	Cerebellum IV-V	CR-IV
Hippocampus	HP	Cerebellum VI	CR-VI
Parahippocampal gyrus	PHG	Cerebellum VIIb	CR-VIIb
Amygdala	AMYG	Cerebellum VIII	CR-VIII
Calcarine	CAL	Cerebellum IX	CR-IX
Cuneus	CUN	Cerebellum X	CR-X
Lingual gyrus	LING	Vermis I-II	VM-I
Superior occipital gyrus	SOG	Vermis III	VM-III
Middle occipital gyrus	MOG	Vermis IV-V	VM-IV
Inferior occipital gyrus	IOG	Vermis VI	VM-VI
Fusiform gyrus	FFG	Vermis VII	VM-VII
Postcentral gyrus	PoCG	Vermis VIII	VM-VIII
Superior parietal gyrus	SPG	Vermis IX	VM-IX
Inferior parietal lobule	IPL	Vermis X	VM-X

### Brain network analysis

Organizational characteristics on functional networks of HF patients and controls were assessed with graph-theoretical analyses [62], using the Brain Connectivity Toolbox (http://www.brain-connectivity-toolbox.net/). We consider brain networks as a graph, G = (N, E), which consists a set of nodes N (brain regions) and connections E (functional connectivity) [31]. Using a threshold of false discovery rate (FDR) < 0.05 [63], we constructed individual brain networks. We considered connected edge weights, if the values were statistically significant, and otherwise set those values to zero and considered not connected. Brain networks were examined for network centrality (degree, strength, and betweenness), network segregation (clustering coefficient and local efficiency), and network integration (nodal efficiency and global efficiency) [31, 32, 62].

Nodal (or regional) degree is defined as the number of connections linking the node to rest of the networks [62], and a brain region showing larger degree values plays a functional core role to highly integrate the multiple specialized functions at different brain regions. Nodal strength, as the weighted degree, is defined as the sum of connection strengths linking the node to rest of the network, and serves as the total level of connection weight information in the node [62]. A larger strength value represents a region that exerts greater connection strength in the communication, and involves a high level of integration in whole-brain communication. Betweenness for a node is measured as the fractional shortest path between any other node pair in the network passing through the node [62]. A brain region showing a higher betweenness value implies that a large number of the shortest path lengths pass through the region, and that the region exerts a high influence in the network communication. The level of functional communication efficiency between any two brain regions can be assumed to be the inverse of the weighted shortest path length, which is the weight sum of connections that must be traversed to travel from one node to another [64]. Weighted nodal efficiency is expressed as the averaged inverse weighed shortest path length to the rest of the network, and global efficiency is defined as the average of all nodal efficiencies [64, 65]. Larger efficiency or shorter path lengths of a region might thus represent that the area communicates more efficiently with the rest of the brain. The level at which a network is organized into densely clustered nodes can be assessed using the clustering coefficient [35]. Weighted clustering coefficient for a region quantifies the number of actual connections existing among the region’s neighbors, proportional to the number of all their possible connections, and a brain region showing a higher weighted clustering coefficient value implies densely linked local structures among the neighboring sites. The segregation index, which is a weighted cluster coefficient, is considered as the weighted shortest path length within the neighbors [62].

### Statistical analyses

The IBM Statistical Package for the Social Sciences (IBM SPSS, v 22, Armonk, NY) software was used to assess demographic, biophysical, and other clinical variables. Demographic, sleep, and other clinical variables were assessed by Chi-square and independent samples t-tests. A threshold value of p<0.05 was considered statistical significance.

We examined FC and graph-theoretical measures between groups using the random permutation test in a nonparametric fashion (p<0.005 for FC, p<0.05 for graph-theoretical measures, 10,000 permutations) [66]. We created a null distribution of t-statistics for each measurement from analysis of covariance (ANCOVA; covariates, age and gender), using group labels randomly-shuffled 10,000 times, with assumption of no significant differences between HF patients and controls. We compared original t-statistic values from ANCOVA with the null distribution, and considered those values significant if they exceeded the distribution threshold. Also, relationships between each variable (BMI, LVEF, PSQI, ESS, BAI, or BDI-II) and FC were examined with partial correlation procedures, with age and gender as covariates (Correlation of BMI, LVEF, PSQI, ESS, BAI, or BDI-II with FC, S1 Fig).

## Results

### Demographic and clinical characteristics

HF patients did not differ in age (p = 0.11) or gender (p = 0.57) compared to controls (Table 1). However, BMI values in HF patients were significantly larger, compared to controls (p = 0.014). Other measurements, including the sleep scores (PSQI, p<0.001; ESS, p = 0.0027), mood values (BDI-II, p<0.001; BAI, p<0.001), and cognitive scores (MoCA, p = 0.01) also showed significant differences between groups (Table 1).

### Whole-brain FC

**Fig 1:**
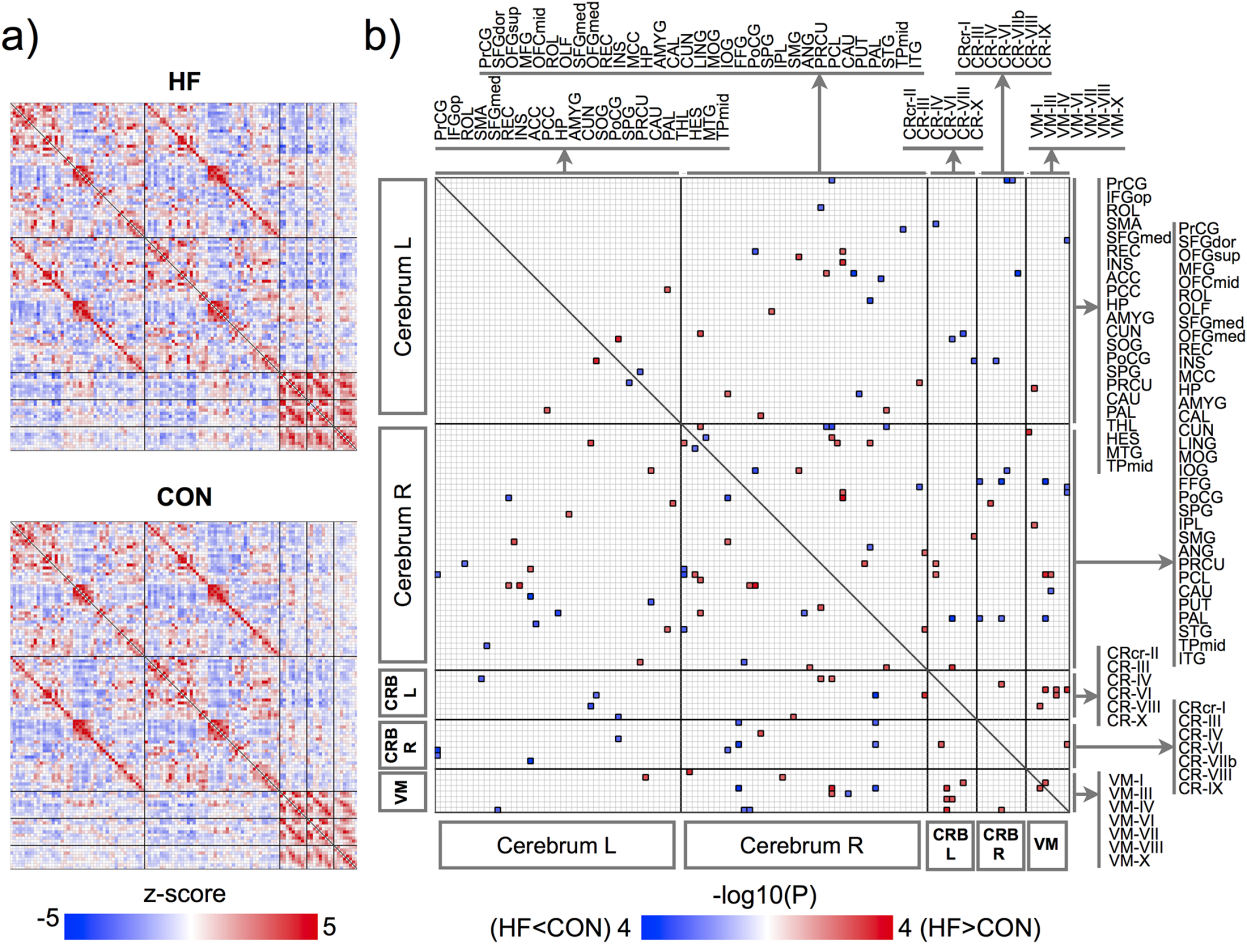
FC matrices for HF and control subjects and group comparison. (a) group-averaging FC matrices for HF and controls. Color bar indicates z-transformed correlation coefficient and red or blue color represents positive or negative FC, respectively. (b) group-comparison FC matrix representing the–log10 (p-values) corresponding to significantly changed FC among all brain site pairs (p<0.005, 10,000 permutations). Red or blue color represents significantly increased or decreased FC in HF, respectively. L and R indicate left and right regions and CRB and VM represent the cerebellum and vermis, respectively. Brain sites with abbreviations are same as listed in Table 2.

**Fig 2:**
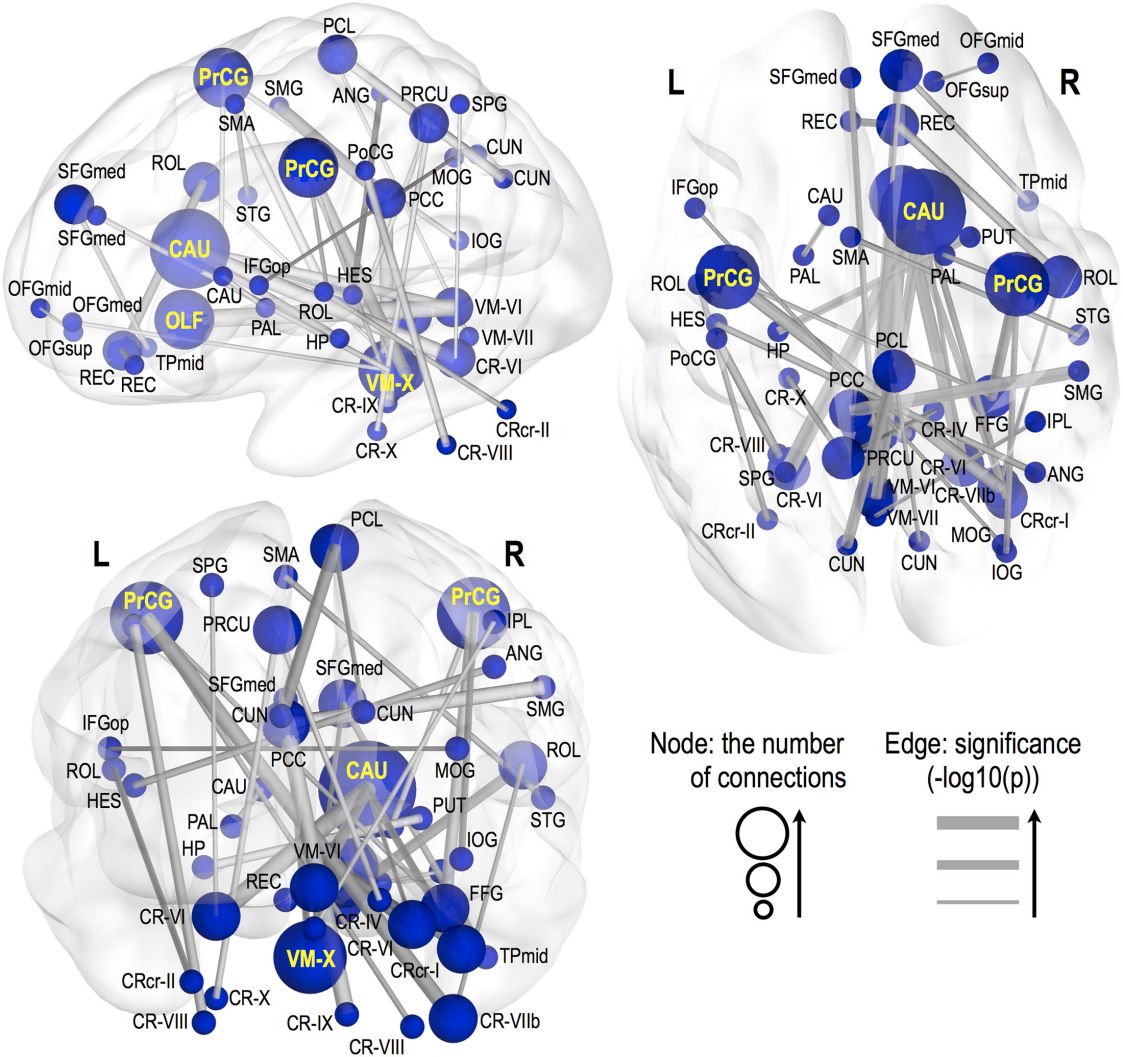
Decreased FC in HF over control subjects. Significantly decreased FC appeared in multiple areas between brain sites in HF patients. Thicker edge lines represent more significant differences, with a scale of–log10 (P-value) from a minimum value to 6, surviving a threshold of p<0.005 (10,000 permutations). Bigger nodal sphere size represents a larger number of significant edges. Bold yellow labels indicate sites with at least 3 functional connections. Brain sites with abbreviations are same as listed in Table 2.

**Fig 3:**
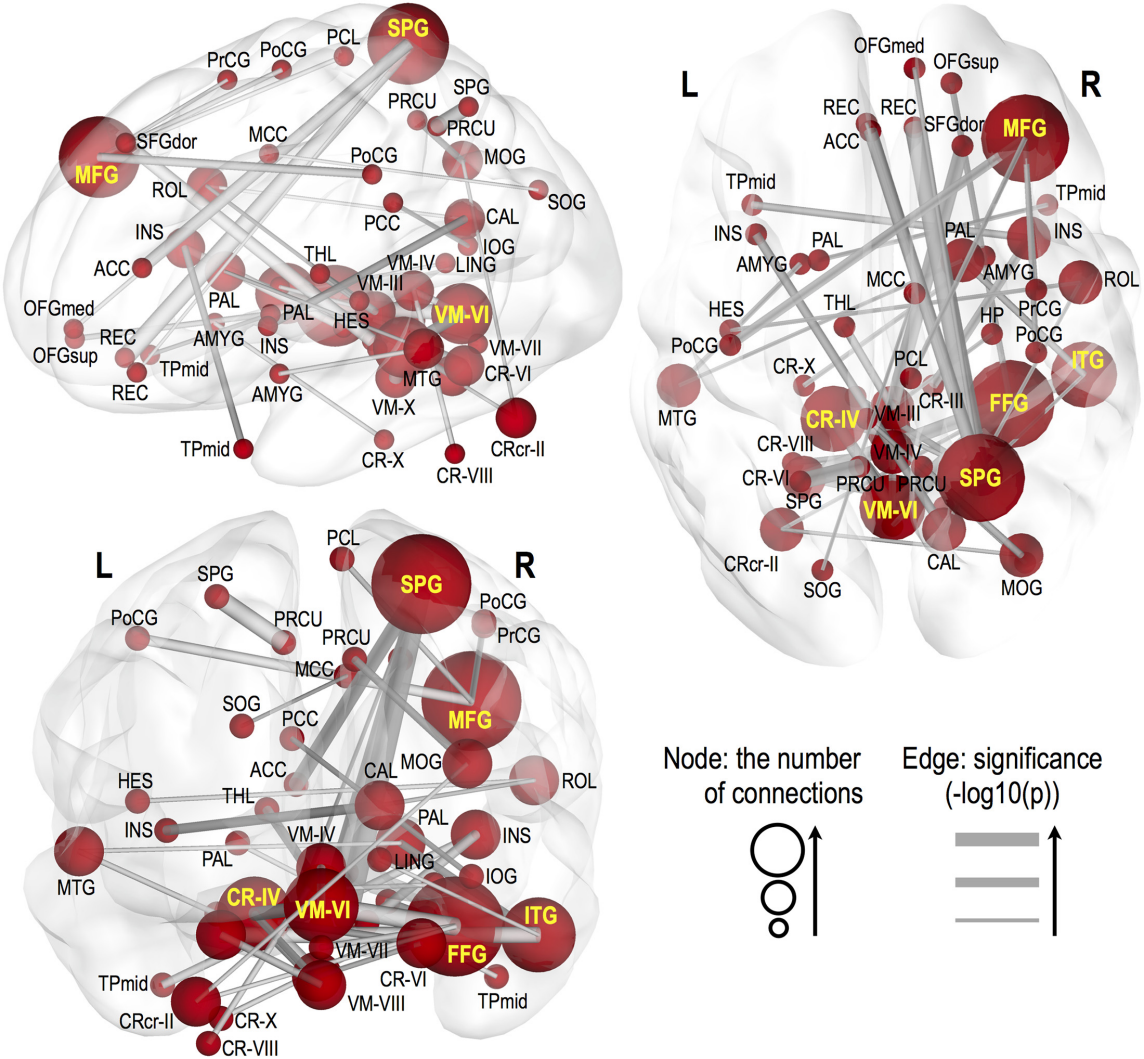
Increased FC in HF over control subjects. Significantly increased FC in areas between brain regions in HF patients. Thicker edge lines represent more significant differences, with a scale of–log10 (P-value) from a minimum value to 6, surviving a threshold of p<0.005 (10,000 permutations). Larger nodal sphere size represents a bigger number of significant edges. Bold yellow labels indicate sites with at least 3 functional connections. Brain sites with abbreviations are same as listed in Table 2.

Significantly altered FC appeared in various brain sites across whole-brain areas in HF, compared to healthy controls (p<0.005, 10,000 permutations), these sites are shown in Figs 1–3, and areas listed in Tables 3 and 4. Decreased FC in HF (Figs 1b and 2), emerged principally between the caudate and cerebellar regions, olfactory and cerebellar sites, vermis and medial frontal regions, and precentral gyri and cerebellar areas. However, increased FC in HF (Figs 1b and 3) appeared between the middle frontal gyrus and sensorimotor regions, superior parietal gyrus and orbito/medial frontal regions, inferior temporal gyrus and lingual gyrus/cerebellar lobe/pallidum, fusiform gyrus and superior orbitofrontal gyrus and cerebellar sites, and within vermis and cerebellar areas, and these connections are largely lateralized to the right hemisphere. Associations between BMI, LVEF, PSQI, ESS, BAI, and BDI-II and functional connections weakly increased or decreased in HF with lower significant level of P<0.05 (r = 0.38, correlation coefficient) (Correlation of BMI, LVEF, PSQI, ESS, BAI, or BDI-II with FC, S1 Fig).

**Table 3:** Significantly decreased FC between brain areas in patients with HF (P<0.005, 10,000 permutations).

Regions	Regions	P-value	Regions	Regions	P-value
Right CAU	Right CRcr-I	0.0021	Right REC	Left REC	0.0017
Right CAU	Left CR-VI	0.0002	Right REC	Right ROL	0.001
Right CAU	Right CR-VI	0.004	VM-X	Left SFGmed	0.0023
Right CAU	VM-VI	0.0008	VM-X	Right SFGmed	0.0038
Left CAU	Left PAL	0.003	VM-X	Right OFGmed	0.0034
Right PUT	Left HP	0.0014	Left PRCU	Right CR-IV	0.0029
Left PrCG	Right FFG	0.0035	Left PRCU	Left CR-X	0.0024
Left PrCG	Right CR-VIIb	0.0004	Left PCC	Right SMG	0.0004
Left PrCG	Right CR-VIII	0.0031	Left PCC	Right CR-XI	0.0003
Right PrCG	Right PAL	0.0029	Right IPL	VM-VII	0.0037
Right PrCG	Right FFG	0.0008	Right ANG	Left HES	0.002
Right PrCG	Right IOG	0.0024	Left SPG	Left CR-VI	0.0039
Left PoCG	Left CR-VIII	0.0012	Left ROL	Left CRcr-II	0.0026
Right PCL	Left CUN	0.0007	Right ROL	Right CR-VIIb	0.0038
Right PCL	Right CUN	0.0038	Right OFGmid	Right OFGsup	0.0045
Right OLF	Right CRcr-I	0.0018	Left IFGop	Right MOG	0.004
Right OLF	Right CR-VI	0.0005	Right SFGmed	Right Tpmid	0.0024
Right OLF	VM-VI	0.0001	Left SMA	Right STG	0.0027

Brain sites with abbreviations are same as listed in Table 2.

**Table 4:** Significantly increased FC between brain sites in patients with HF (P<0.005, 10,000 permutations).

Regions	Regions	P-value	Regions	Regions	P-value
Right MFG	Right PrCG	0.0044	Left PRCU	Left SPG	0.0004
Right MFG	Left PoCG	0.0015	Right PRCU	Right MOG	0.0024
Right MFG	Right PoCG	0.0033	Right ROL	Right CAL	0.0046
Right MFG	Right PCL	0.0036	Right ROL	Left HES	0.0043
Right SPG	Right OFGmed	0.0038	Right MCC	Left SOG	0.0049
Right SPG	Left REC	0.0049	Left PCC	Right IOG	0.0038
Right SPG	Right REC	0.0002	Right AMYG	Left CR-X	0.0037
Right SPG	Left ACC	0.0007	Left AMYG	Left MTG	0.0036
Right FFG	Right OFGsup	0.0029	Right HP	VM-III	0.0043
Right FFG	VM6	0.0004	VM-III	Left THL	0.0019
Right FFG	Left CRcr-II	0.0044	Left CRcr-II	Right MOG	0.0045
Right FFG	VM-VII	0.0033	VM-I	Right SFGdor	0.0006
Right ITG	Right LING	0.0048	Right CR-VI	Left CR-III	0.0028
Right ITG	Left CR-VI	0.0006	Right CR-VI	VM-X	0.0039
Right PAL	Right ITG	0.0026	Left CR-IV	VM-VI	0.0009
Right PAL	Left MTG	0.0045	Left CR-IV	VM-VIII	0.0027
Left PAL	Right Tpmid	0.004	Left CR-IV	VM-X	0.0005
Right INS	Left Tpmid	0.0022	VM-IV	Left CR-VIII	0.0032
Right INS	Right CR-III	0.0017	VM-IV	VM-VI	0.0022
Left INS	Right CAL	0.0017	VM-VIII	Left CR-VI	0.0025

Brain sites with abbreviations are same as listed in Table 2.

#### Decreased FC in HF

Both the left and right precentral gyrus showed decreased FC in HF with regions in the right hemisphere, i.e., the right cerebellar lobe VIIb/VIII and fusiform gyrus connected with the left precentral gyrus and the right pallidum, fusiform gyrus, and inferior occipital gyrus connected with the right precentral gyrus. Connectivity between the left postcentral gyrus and left cerebellar lobe VIII was also diminished in HF. The right paracentral lobule showed decreased connections with the bilateral cuneus, and the right olfactory with the cerebellar regions, including the right crus I, right lobe VI, and vermis VI. The right rectus showed reduced FC with the left rectus and right Rolandic operculum, and with right middle and superior orbitofrontal gyri. The vermis X showed decreased FC with the bilateral medial superior frontal and right medial orbitofrontal gyri. The right caudate remarkably showed decreased FC with the cerebellar sites, including the right crus I, bilateral lobe VI, and vermis VI. However, the left caudate showed reduced FC with the left pallidum only. Also, decreased FC emerged between the cerebellar and posterior parietal regions, including the posterior cingulate cortex, precuneus, inferior parietal lobule, and supramarginal gyrus.

#### Increased FC in HF

The right middle frontal gyrus showed increased FC with the bilateral postcentral gyri, right precentral gyrus, and right paracentral lobule. Several sites within-cerebellar areas emerged with increased FC between the vermis VI and the vermis IV, and between left cerebellar lobe IV-VI and the vermis VIII-X. The right superior parietal gyrus showed increased FC with the bilateral rectus, right medial orbitofrontal gyrus, and left anterior cingulate cortex. The right insula showed increased FC with the left middle temporal pole and right cerebellar lobe III, while the left insula with the right calcarine. Connections between the right amygdala and left cerebellar lobe X, the left amygdala and the left middle temporal gyrus, the right hippocampus and vermis III, and the left thalamus and vermis III appeared with increased FC. In addition, the right fusiform gyrus showed enhanced FC with the right superior orbitofrontal gyrus, left cerebellar crus II, and vermis VI-VII. However, the right inferior temporal gyrus showed increased FC with the right lingual gyrus, left cerebellar lobe VI, and right pallidum.

### Topological measures

**Fig 4:**
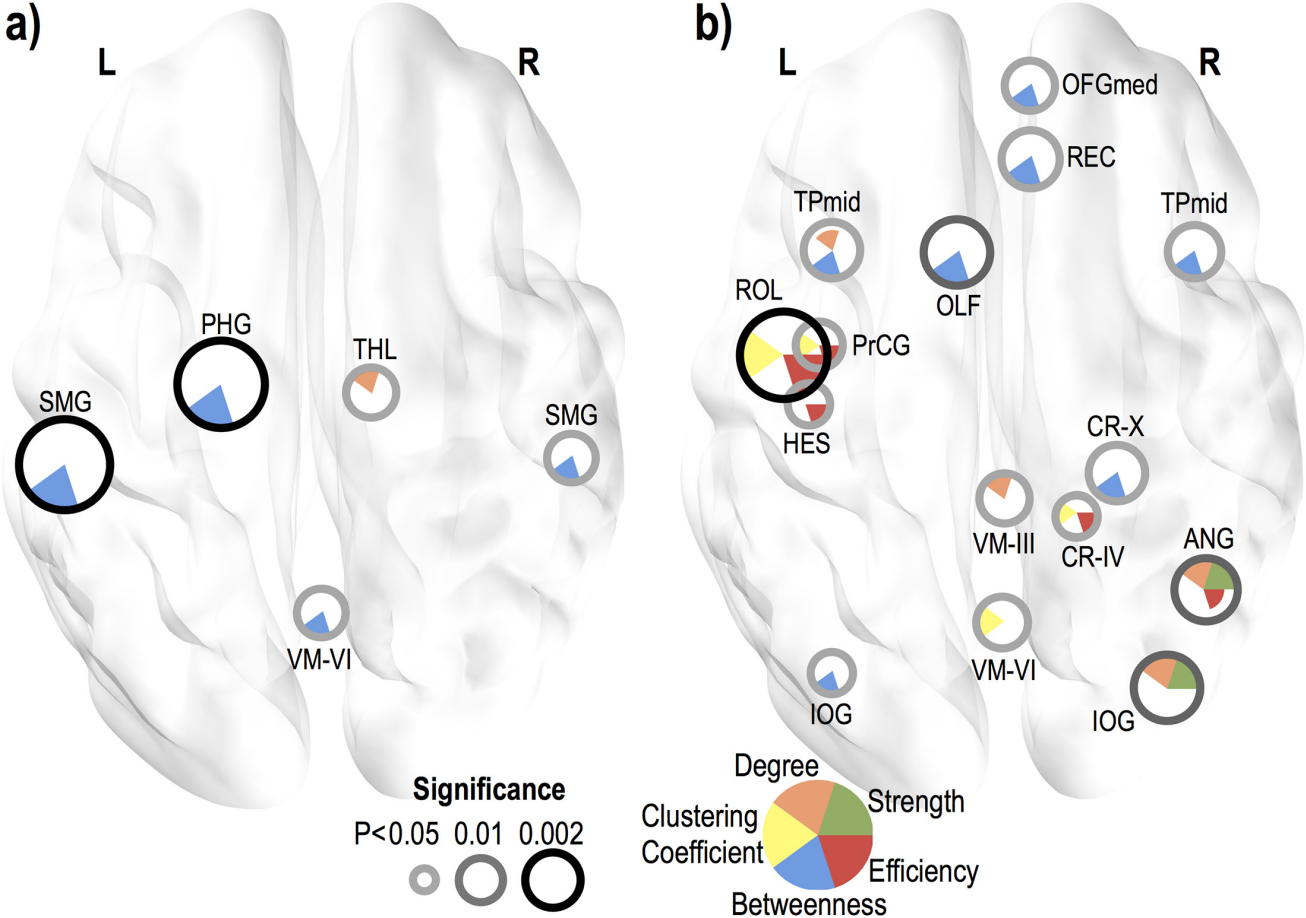
Graph-theoretical measures in HF subjects. Significantly decreased (a) or increased (b) graph-theoretical measures in HF subjects (p<0.05, 10,000 permutations). Each circle represents significantly changed regional (or nodal) properties, with various color representing strength, degree, weighted clustering coefficient, betweenness centrality, and nodal efficiency, respectively. Brain sites with abbreviations are same as listed in Table 2.

Nodal topological measures in HF showed altered values in widespread brain regions (Fig 4, Table 5; p<0.05, 10,000 permutations). Network centrality measures in HF appeared with decreased betweenness centrality at the left para-hippocampal gyrus, left and right supramarginal gyrus, and vermis VI, and decreased degree at the right thalamus. Increased betweenness centrality emerged at the right medial orbitofrontal gyrus, right rectus, left olfactory, bilateral middle temporal pole, left inferior occipital gyrus, and right cerebellar lobule X. However, increased degree appeared at the left middle temporal pole, right angular gyrus, right inferior occipital gyrus, and vermis III, and increased strength at the right angular gyrus and right inferior occipital gyrus. HF subjects showed increased weighted clustering coefficient at the left precentral gyrus, left Rolandic, right cerebellar lobule IV-V, and vermis VI, and increased nodal efficiency at the left precentral, left Rolandic, left heschl, right angular gyrus, and cerebellar lobule IV-V. Both weighted clustering coefficient and nodal efficiency did not show any site with decreased value in HF. Also, global network properties, including global and local efficiency, did not show any significant difference.

**Table 5:** Significantly different nodal properties in HF patients (P<0.05, 10,000 permutations). Negative and positive p values correspond to decreased and increased value in HF, respectively.

Regions	Hemisphere	Betweenness	Degree	Strength	Weighted clustering coefficient	Efficiency
OFGmed	Right	0.0271	−	−	−	−
REC	Right	0.0139	−	−	−	−
OLF	Left	0.0079	−	−	−	−
SMG	Left	-0.0004	−	−	−	−
SMG	Right	-0.0238	−	−	−	−
PrCG	Left	−	−	−	0.0264	0.0351
Tpmid	Left	0.0165	0.0495	−	−	−
Tpmid	Right	0.0277	−	−	−	−
ROL	Left	−	−	−	0.0015	0.001
HES	Left	−	−	−	−	0.0421
ANG	Right	−	0.0163	0.0096	−	0.0493
IOG	Left	0.0481	−	−	−	−
IOG	Right	−	0.0099	0.0113	−	−
THL	Right	−	-0.0358	−	−	−
PHG	Left	-0.0018	−	−	−	
CR-IV	Right	−	−	−	0.0439	0.0439
CR-X	Right	0.012	−	−	−	−
VM-III	−	−	0.0295	−	−	−
VM-VI	−	-0.0215	−	−	0.0204	−

Brain sites with abbreviations are same as listed in Table 2.

## Discussion

We examined whole-brain FC and their network organizational properties in HF patients compared to controls using rs-fMRI procedures. A core question was how brain dysfunction in HF condition affects individual functional interactions and coordination among sites across the whole-brain. Our data suggest that HF patients have aberrant spontaneous functional connections in various brain areas, especially lateralized to the right hemisphere. These aberrant connections are related to sensorimotor, autonomic, mood, and cognitive regulation, sites which have been reported as having structural injury or being deficient in function when challenged in previous HF studies. Moreover, functional interactions altered in HF contribute to aberrant brain network organization in the condition.

### Functional reorganization in autonomic, respiratory, and sensorimotor networks

Autonomic dysfunction, including increased sympathetic tone, aberrant heart rate, and blood pressure responses to cardiovascular challenges, is a core characteristic in HF [3, 4, 67, 68]. Damaged brain structures in HF include the insular, cingulate, orbitofrontal, hypothalamus, and cerebellar regions [13–16].

The right and left insular cortices exert influences on sympathetic and parasympathetic nervous system activity [69–71] and both receive visceral sensory input from, and project to, the hypothalamus, participating significantly in autonomic regulation [71]. In addition, the insular cortices play significant roles in pain mediation [72], and in dyspnea [73], both issues of concern in HF. The cingulate cortex, which receives axons from and projects to insular cortices, mediates both autonomic sympathetic and parasympathetic branches, and damage to this structure can impact cardiac regulation [3, 15, 74, 75]. The orbitofrontal cortex exerts prominent influences on somatomotor inhibition of autonomic responses, and coordination of behavioral responses during adaptation [76] and shows a substantial role in initiation of blood pressure responses [77]. The cerebellar cortices and vermis play autonomic and respiratory motor regulation [78, 79], and are one of the heavily damaged regions in HF [13–16], and also show altered autonomic responses to cardiovascular challenges [3, 4].

Over the regional FC changes, our findings show that the injury in these autonomic and respiratory control sites further leads to decreased functional interactions among these regions and/or with other cognitive control regions (e.g., the bilateral medial superior frontal gyri). Notably, such declines in FC are primarily localized in the right hemisphere, and the right olfactory and vermis X were sites to play key roles. The functional lateralization in HF may couple with lateralized tissue damage in the condition, which consistently appeared in the previous studies [13, 15, 16]. Increased functional interactions with the bilateral insula and increased within-cerebellar regions in HF may correspond to enhanced local plasticity of fibers, such that additional regions are recruited for the diverse functional compensatory processes. Such processes are necessary to protect against abnormalities that appear in HF, consequences of vulnerable regulation by exaggerated sympathetic outflow or metabolic stress [80].

Reorganization of FC in autonomic and respiratory systems in HF subjects may also underlie deficient sensorimotor processes, reflecting distorted sensory input from the upper airway, and contributing to the atonia in upper airway muscles during inspiratory efforts of obstructed breathing in HF. Here, distorted sensorimotor integration appeared as decreased FC largely involved in the bilateral precentral gyri, as well as in the right paracentral lobule and left postcentral gyrus. Moreover, these decreased connections are largely lateralized in the right hemisphere, similar to the declines found in autonomic regulatory sites.

### Functional reorganization in neurocognitive networks

HF patients show many cognitive issues, including affective, executive, memory, attention, behavioral, and learning functions [81]. One of the remarkable outcomes that emerged was the appearance of several lateralized abnormal connections anchored at the right caudate and middle frontal gyrus, which presumably contribute to executive deficits in the condition. We speculate that injury in autonomic and respiratory regulatory cerebellar regions may serve decreased functional connections with the right caudate, eventually leading to executive, behavioral, and learning deficits in HF, and reducing the well-known contributions of the basal-ganglia to autonomic regulation [82]. Similarly, damaged sensorimotor regions may also contribute to such functional deficits by abnormally increased (compensatory) interactions with the right middle frontal gyrus.

Other symptoms in HF include an increased incidence of mood disorders [12], which presumably result from regional injury in the prefrontal cortex, para-hippocampal gyrus, cingulate, insula, hippocampus, and cerebellum [15]. In this study, the right amygdala, right hippocampus, and left thalamus (as well as the bilateral insula) in HF showed increased FC with cerebellar sites. These regions reveal exaggerated sympathetic outflow or high metabolic demand, which presumably result from a compensatory mechanism by engaging additional regions with enhanced functional connections. Thus, it may be the case that injury in autonomic and respiratory regulatory cerebellar regions might contribute to increased connections with brain sites serving as high level of mood control in HF. Meanwhile, increased FC between the right hippocampus and vermis III may also contribute to memory loss in HF [15, 83]. The fusiform gyrus is connected with several cerebellar regions, and may affect processing of imaginative fearful objects of anxiety aspects in HF [84].

Deficient processing in attention is common in HF [81], and may be associated with the abnormal network coordination from the posterior parietal cortex (e.g., the posterior cingulate cortex, precuneus, and superior/inferior parietal regions), as observed in our study [85, 86]. Also, the right superior parietal gyrus was observed as a core region, collecting increased connectivity with anterior brain sites within autonomic and respiratory regulatory circuitry. The posterior cingulate cortex and precuneus showed decreased FC with the cerebellum, but showed increased FC with the occipital areas. These findings may suggest mechanisms for the impaired attention and visual processing in HF through abnormal connections with the posterior parietal cortex in the condition [85–87]. Moreover, abnormal FC from the posterior cingulate cortex could contribute to depressive symptoms in the condition [88].

### Alterations in topological attributes

Human brain functions are represented by various configurations between local specialization and global integration among brain regional activities [31, 32]. Examining brain network organizational abnormalities can thus provide new insights in exploring disease pathology [89]. Declined regional metabolism within brain tissues and synaptic injury in a disease group may result in disrupted anatomical projections, alter FC, and eventually give rise to an abnormal functional brain network pattern emerging as less effective and reduced regional centrality in core brain areas (with a compensatory increase in other regional central sites), as shown in Alzheimer's disease [90, 91], Parkinson’s disease [92], and Stroke [93]. Network-level analyses using graph theory in a disease group serve a research framework to explore brain network organization with topological properties [31, 32].

In this study, HF patients showed, across broad regions, predominantly increased topological properties that may result from increased metabolic activity in HF. Increased regional centrality was localized in autonomic and respiratory regions, and bilateral middle temporal pole, inferior occipital gyrus, and right angular gyrus, which may underlie known cognitive issues in the condition. The weighted clustering coefficients (e.g., a measure of regional segregation) and regional efficiency (e.g., an integration measure between two sites in neural information delivery) in HF patients were significantly increased in the precentral, temporal, cerebellar, and right angular regions. Increased regional centrality, segregation, and efficiency of brain networks in HF indicate brain areas unexpectedly engaged by compensatory coordination in the condition, which may represent an exaggerated hub or integrative role for the flow of brain information. Reduced regional centrality in the left para-hippocampal gyrus, bilateral supramarginal gyrus, right thalamus, and vermis VI could contribute to cognitive deficits in HF. Both the para-hippocampal and supramarginal regions are involved in higher-order cognitive/behavioral functions [94], and in integration and interactions involving visual, auditory, and somato-sensory functions with adjoining sensory regions [95], respectively. Reduced regional centrality of brain networks in HF shows a diminished hub role for the flow of brain information.

### Potential pathological processes

Our findings show that HF brain is not simply affected by localized injury, but also accompanied by abnormal functional network coordination among such damaged areas that serve many of the autonomic, sensorimotor, and cognitive functions deficient in the condition. Several pathological processes may contribute to abnormal functional network properties, including low cardiac output [3] and hypoxia/ischemia processes from sleep disordered breathing issues [96], leading to cerebral perfusion issues. Both processes may result to localized cortical changes, mainly at cortical hub regions. Also, injury to autonomic regions may alter vascular supply to other cortical sites that may induce secondary damage to other brain areas across the brain, and eventually may result in abnormal functional brain network in the condition.

### Limitations

Several limitations of this study should be acknowledged. We evaluated individual brain networks by splitting the whole-brain into 116 regions, as a widely used parcellation scheme in brain network studies. Further studies are required to compare the current findings using different parcellation schemes, since their uses could exhibit different graph-theoretical results, based on variable regions of interest [97–100]. Also, all subjects were instructed not to focus on any specific thoughts during scanning, we could not ensure about this issue, and could be considered as potential limitation. However, heart rate was carefully monitored in all subjects during the resting-state functional MRI, and none of subjects included here showed significant heart rate fluctuation, indicating that subjects followed our instruction for not focusing on specific thoughts during the resting-state functional MRI.

## Conclusions

Heart failure patients show resting-state spontaneous brain dysfunction between multiple sites, and autonomic, cognitive, and affective deficits may stem from the altered FC and brain network organization. The altered FC in HF is largely lateralized to the right hemisphere, which may result from previously-identified lateralized tissue changes in the condition. These increased and decreased FC deficits may contribute to higher morbidity and mortality in the condition. The adverse clinical outcomes likely result from the prominent structural changes in both axons and nuclear structures reported earlier in HF; protecting neural tissue may improve functional network integrity, and thus, reduce morbidity and mortality and increase quality of life in the condition.

## Supporting Information

S1 Fig Correlation of BMI, LVEF, PSQI, ESS, BAI, or BDI-II with FC. Weak relationship trend between each variables and functional connections. Blue and red color represents negative and positive relationships, respectively. Other figure conventions are same as in Figs 2 and 3.
(DOCX)
