# Supplementary material for: Lateralized Resting-State Functional Brain Network Organization Changes in Heart Failure
Source: PLoS One. 2016 May 20;11(5):e0155894. doi: 10.1371/journal.pone.0155894 (PMC4874547; doi:10.1371/journal.pone.0155894)
Supplement: S1 Fig — Weak relationship trend between each variables and functional connections. Blue and red color represents negative and positive relationships, respectively. Other figure conventions are same as in Figs 2 and 3. (DOCX) [file pone.0155894.s001.docx]

**Supplementary Material**

We examined relationships between each variable (BMI, LVEF, PSQI, ESS, BAI, or BDI-II) and functional connections found as just weakly increased or decreased in HF subjects. Each relationship was examined by partial correlation analysis, with covariates included as age and gender. As pathological trends, we report the relationships with weak significant level of P<0.05 (r=0.38, correlation coefficient), even if we did not find any strong relationship with the same significant level of P<0.005.

*
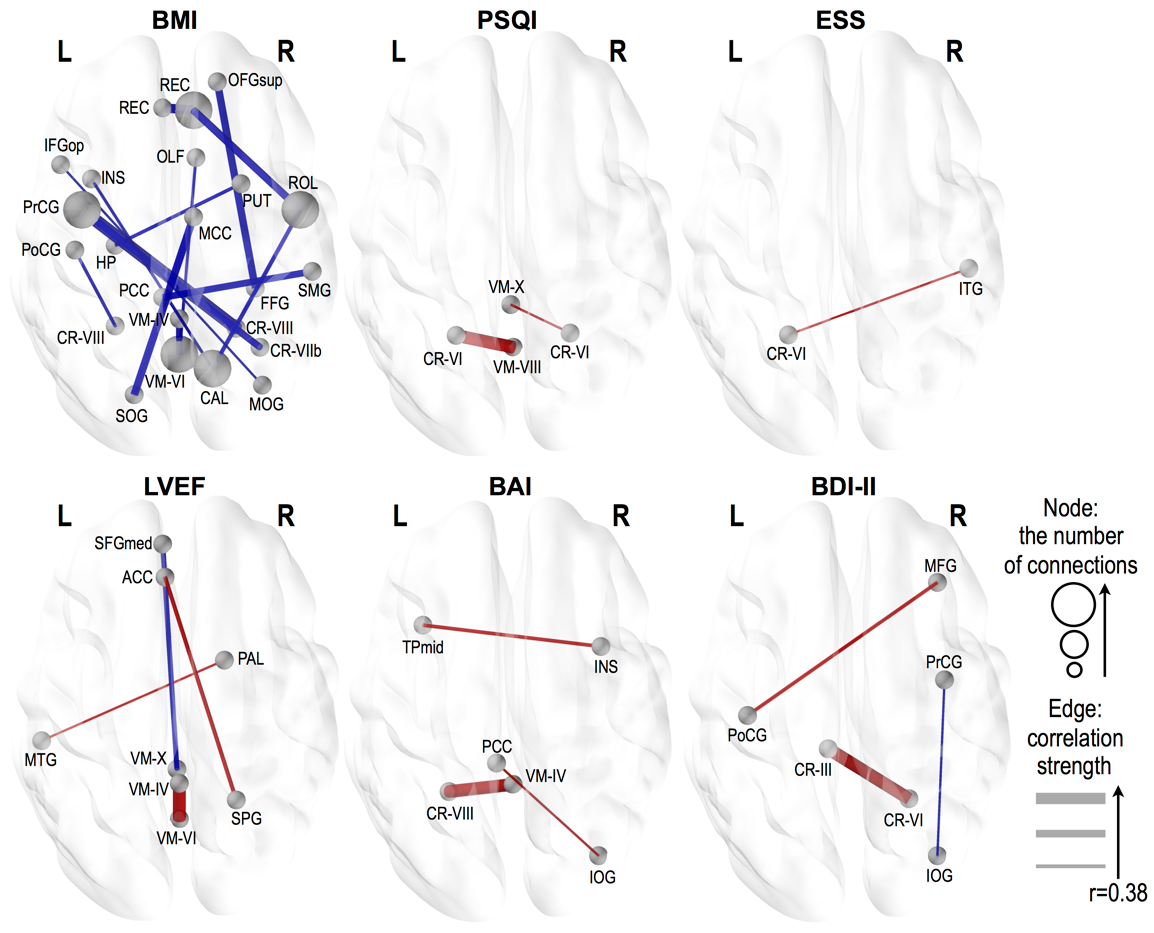
*

**S1 Fig.** **Correlation of BMI, LVEF, PSQI, ESS, BAI, or BDI-II with FC.** Weak relationship trend between each variables and functional connections. Blue and red color represents negative and positive relationships, respectively. Other figure conventions are same as in Figures 2 and 3.
